# Drivers of Pancreatic Cancer: Beyond the Big 4

**DOI:** 10.3390/cancers17142354

**Published:** 2025-07-15

**Authors:** Laura M. Porcza, Rafael Ballesteros-Cillero, Lok To Lam, Cristina Maiello, Nicholas R. Leslie

**Affiliations:** Institute of Biological Chemistry, Biophysics and Bioengineering, Heriot-Watt University, Edinburgh EH14 4AS, UK; l.porcza@hw.ac.uk (L.M.P.); cristinamaiello508@gmail.com (C.M.)

**Keywords:** pancreatic cancer, mutation, protein, immunohistochemistry, PTEN

## Abstract

**Background:** Pancreatic cancer is frequently identified as the cancer type with the shortest probable survival time after diagnosis, and efforts to develop successful treatments have had a very limited impact in the clinic. One reason for the limited therapeutic options is the lack of appealing drug targets. The great majority of pancreatic cancers are classified as Pancreatic Ductal Adenocarcinoma (PDAC), in which the genetic landscape is dominated by four genes: *KRAS*, *TP53*, *CDKN2A*, and *SMAD4*. However, despite extensive knowledge of these genetic drivers, the development of effective therapies has seen only very limited success. **Methods**: Existing evidence indicates that mutations in the tumour suppressor gene *PTEN* are uncommon in PDAC (<10% cases). However, the loss of PTEN function through non-genetic mechanisms may be much more common and have a strong impact. We therefore summarise and review a large body of immunohistochemical studies that address the loss of PTEN in PDAC as well as a smaller number of studies addressing other implicated proteins, including KDM6A and ARID1A. **Results**: These studies show some loss of PTEN protein in more than half of PDAC cases. Furthermore, although genetic changes in genes including KDM6A/UTX and ARID1A are also uncommon, reduced expression of their encoded proteins is observed in many, perhaps most, cases of PDAC. **Conclusions**: These analyses, which go beyond genetics, highlight the broader set of cellular functions that are dysregulated in many pancreatic cancers and provide broader opportunities for treatment strategies. This review highlights the emerging importance of other drivers in PDAC, which are less well-studied in this context.

## Simple Summary

Cancer is a complex disease influenced by many factors that determine how genetic changes affect the function of encoded proteins, ultimately driving cancer development. While much pancreatic cancer research focuses on the four most frequently mutated genes (KRAS, TP53, CDKN2A, and SMAD4), this review highlights the significant, but less-understood roles of further tumour suppressors, such as PTEN, KDM6A, and ARID1A in PDAC. Mutations in these genes are uncommon, but a reduced expression of their proteins is often observed in tumours. We present summaries of this data and emphasise the importance of research beyond the “big four” genes to better understand and combat this aggressive type of cancer.

## 1. Introduction

More than 80% of primary cancers identified in the pancreas are defined as Pancreatic Ductal Adenocarcinoma (PDAC). This represents one of the worst diagnoses amongst all malignancies, with recent data showing only 12% of patients surviving 5 years after diagnosis in the USA, with a median survival of around 6 months [1]. This is a very modest improvement on older data (which remain frequently reported); however, many parts of the world still see 5 year survival well below 12% [1]. Combined with a relatively high incidence, this results in pancreatic cancer now being the third most common cause of cancer death in the USA and Europe, after lung and colorectal cancer [1,2]. One factor in this poor prognosis is that diagnosis is commonly made only at the stage of advanced disease due to a lack of specific symptoms and tumour markers. Frequently, local invasion and distant metastasis occur prior to diagnosis, significantly lowering the possibility of curative surgery. Furthermore, PDAC very rarely responds well to chemotherapy or radiotherapy, and as will be discussed, there is a lack of effective targeted biological therapies [3]. A key histological feature of PDAC, which may contribute to its therapeutic resistance, is its unusually fibrous stroma. This desmoplasia is even observed in relatively early-stage tumours [4,5]. Addressing this point, a well-defined set of precursor lesions has been identified, which is believed to have the potential to progress into PDAC. These include changes generally termed Pancreatic Intraepithelial Neoplasia (PanIN), which are frequently subdivided into low-grade and high-grade lesions [6].

There are several less common types of cancer in the pancreas, including neuroendocrine tumours and acinar carcinomas. However, these will not be discussed in any detail in this review, except where relevant clinical studies mention pancreatic cancer without specifying whether the patients and samples are restricted to PDAC or also include other types of pancreatic cancer. This review will therefore focus specifically on PDAC, highlighting the significant, but less-understood roles of tumour suppressors, such as PTEN, KDM6A, and ARID1A in PDAC.

## 2. Genetics of Pancreatic Ductal Adenocarcinoma

Genomic analyses of pancreatic cancer have consistently identified a well-recognised set of recurrent mutations, which are present in many, and in some studies the majority of these tumours [7,8,9,10,11,12,13]. In particular, coding sequence variants in *KRAS*, *TP53*, *CDKN2A*, and *SMAD4* have been identified in all such studies, in most cases emerging as the four most commonly mutated genes. Most notably, activating missense mutations in *KRAS* are identified in almost all cases of PDAC, with observed rates in most patient cohorts >90% [7,8,9,10,11,12,13,14,15]. *KRAS* mutations also appear to be a very early, probably initiating event in PDAC development, implied by their frequent discovery in PanIN lesions [16]. Furthermore, most PDAC cases also display loss-of-function mutations in TP53, with cohorts commonly showing rates in the 60–75% range. There appears to be less consistency in the observation of changes in the *CDKN2A* and *SMAD4* tumour suppressor genes, with estimates for their functional loss ranging from 15% to 60%, perhaps contributed by different assessment technologies and differences between the analysed populations [7,8,9,10,11,12,13,14,15]. While there is broad agreement that a KRAS mutation is an early and likely initiating event in PanIN/PDAC development, the sequence of subsequent genetic alterations remains less well understood. *CDKN2A* loss of function is often presented as a subsequent event during the progression through the stages of PanIN, while changes in *TP53* and *SMAD4* only occur later in well-developed PanIN lesions and during the progression to PDAC [17,18]. Therefore, an in-depth understanding of the evolution of PDAC is yet to be achieved, with particular attention being paid to whether mutations accumulate gradually or in a punctuated manner, as well as to how the cancer cell genetic landscape interacts with the tumour microenvironment [6,18].

Given the central role of KRAS, TP53, CDKN2A, and SMAD4 in PDAC, we briefly summarise their biological functions and implications for tumour development. *KRAS* (Kirsten rat sarcoma virus) encodes the small-GTPase KRAS, which acts as an evolutionarily conserved switch-like activator of mitogenic and growth signalling in animal cells [19,20]. In the context of PDAC, one of the most significant pathways through which KRAS drives tumour progression is via the activation of the class 1 phosphoinositide 3-kinase (PI3K) enzymes and their synthesis of the lipid signal PIP_3_, which is removed by the tumour suppressor PTEN [19,20]. The *TP53* tumour suppressor gene encodes the p53 transcription factor, which activates specific programmes of gene expression in response to cell stresses, including DNA damage. The responses activated by p53 target genes include cell cycle arrest, DNA repair, metabolic reprogramming, and depending on the context, cell death [21]. Unusually, the *CDKN2A* (cyclin-dependent kinase inhibitor 2A) gene encodes two unrelated proteins with independent tumour suppressor functions, p14 ARF and p16 INK4A. The ARF protein is a key activator of p53 tumour suppressor function, which acts by protecting p53 from degradation, and the INK4A protein blocks the promotion of cell proliferation by the cyclin-dependent kinases CDK4 and CDK6 [22]. Finally, a significant minority of PDACs contain mutations in the *SMAD4* gene, also sometimes referred to as Deleted in Pancreatic Cancer-4 (DPC4), with such mutations being less common in other types of cancer. The *SMAD4* gene encodes a transcription factor that drives many of the effects of the intercellular signal protein Transforming Growth Factor beta (TGFβ). The SMAD4 protein forms hetero-oligomers with other SMAD proteins in response to TGFβ stimulation and, through effects on the transcription of its target genes, influences a diverse set of processes including cell cycle progression, cell migration, and angiogenesis [23].

A notable further feature revealed by these genomic studies is the relative homogeneity of these tumours, which are defined as pancreatic cancer, when compared to cancers identified in other organs. Cancers in almost all other organ systems display multiple clearly distinct cancer types, which themselves display greater apparent heterogeneity within their driving mutations, when compared to the picture observed in pancreatic cancer cohorts. These studies of pancreatic cancer also suggest that, despite a relatively homogeneous mutational landscape dominated by a few key drivers, additional genetic and non-genetic alterations contribute to PDAC heterogeneity and progression.

**Figure 1:**
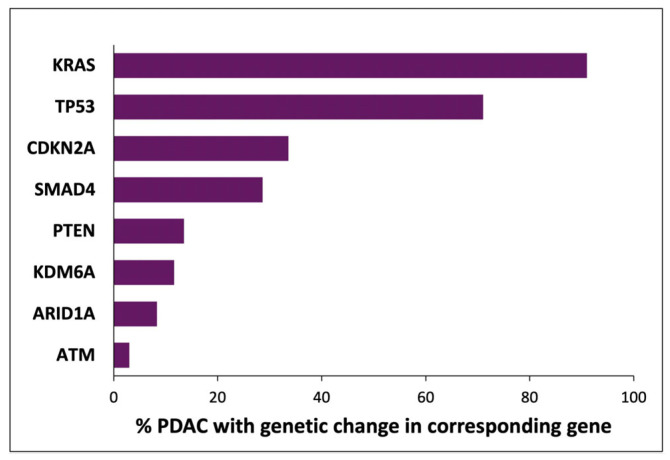
Indicative fractions of PDAC cancers with genetic changes in selected genes. The frequency of genetic changes in each gene was calculated as the mean from multiple studies [7,8,9,10,11,12,13,14,15]. The analyses and methods in these reports are not uniform, and the presented values represent an amalgamation of multiple classes of genetic changes. The genes discussed in this review and mutated in < 15% cases (PTEN, KDM6A, ARID1A, and ATM) are shown for relevance and were selected from a large number of genes (approx. 20) in which genetic changes were reported in 3–15% of PDACs.

The consistent nature of less frequent genetic changes in PDAC indicates that additional genes also represent driver oncogenes and tumour suppressors (see Figure 1) [7,8,9,10,11,12,13,14,15]. For example, almost all of the changes in *MYC* identified in around 5% of PDAC cases are amplifications, while almost all mutations of *ARID1A* present in 5–15% of PDAC cases are truncations [8,11,13,24]. This list of genes in which genetic changes are less frequently identified may indicate that functional changes in the relevant encoded proteins occur only through genetic mechanisms and only in a small subset of PDAC cancers. However, it is also possible that the function of these proteins is significant and modified in relatively many cases of PDAC, but by mechanisms not detected in these genomic studies.

For context, as mentioned above, genetic changes in *CDKN2A* and *SMAD4* are each found in approximately one in three cases of PDAC [7,8,9,10,11,12,13,14,15]. Immunohistochemical analyses of the expression of the encoded proteins (the SMAD4 protein and the p16 INK4A and p14 ARF proteins encoded by the *CDKN2A* locus) report loss of SMAD4 protein expression in approximately 55% of PDAC samples [25,26,27,28,29,30,31] and loss of p16/INK4A and p14/ARF in a slightly higher fraction of cancers. Rates of reported loss of p16/INK4A (approx. 70%; range: 46–100%) are similar to those for p14/ARF (two studies reporting 65% and 68%) [25,26,27,28,29,30,31,32]. Therefore, for both CDKN2A and SMAD4 loci, the frequency at which loss of protein expression is reported (Table 1) is approximately double the reported frequency of mutation, indicating common non-genomic mechanisms by which protein function is lost.

## 3. Can PDAC Genetics and Stratification Inform Successful Therapy?

The last ten to fifteen years have seen substantial efforts to identify predictive genetic biomarkers for targeted therapy [33]. There have been some successes reported, including increased progression-free survival in germline BRCA mutant patients with metastatic PDAC treated with the PARP inhibitor Olaparib [34]. However, such BRCA mutations are present only in a small fraction of PDAC patients (approx. 5%) and consistent benefits on overall survival have not yet been seen [34,35], leaving the best use of these drugs in PDAC to be determined [36,37]. Success with the use of the novel KRAS inhibitor Sotorasib in lung cancer patients and colon cancer patients with cancers harbouring specifically G12C mutant KRAS protein [38,39] has raised hope for similar treatments for PDAC. The KRAS G12C mutation is rare in PDAC (approximately 1% of patients), and the selectivity of Sotorasib and other KRAS G12C selective inhibitors appears to rely on the presence of the novel cysteine residue in the switch II region close to the nucleotide-binding site of KRAS, requiring novel strategies to develop drugs targeting other KRAS mutants, which are more common in PDAC [40]. Despite these challenges, a number of inhibitors targeting KRAS G12D have emerged and are showing promise in early clinical trials [41,42].

In recent years, several efforts have been made to identify subtypes of PDAC to understand the disease better and to improve prognosis and therapy. These studies have revealed some consistent patterns, with multiple genetic and gene expression analyses aligning somewhat to identify PDAC subtypes. One subtype, often termed ‘classical,’ exhibits more epithelial and adherent characteristics, while an alternative subtype, referred to as ‘quasi-mesenchymal,’ ‘basal-like,’ or ‘squamous’, displays more mesenchymal traits [13,43,44,45]. However, consensus has yet to be reached regarding the relationship of this classification to further subtypes, associations with stromal or immune cell types, and chromosomal structural changes, as well as cancer morphology and histology [46,47]. Nevertheless, as the volume and quality of PDAC cohort data continue to grow alongside advancements in analytical techniques, improved outcomes for more patients are likely to follow.

The field of immune therapy for cancer has yielded many successes in the last fifteen or so years [48], and efforts have been made to apply immune therapy to pancreatic cancer. Furthermore, PDAC-specific therapeutic strategies have also been developed to target other non-immune aspects of the tumour microenvironment. Initial efforts have been largely unsuccessful [49]; however, an emerging understanding of the positive and negative impacts of the tumour stroma [50,51], intratumoural heterogeneity [52,53], and links to tumour genetics and subtypes should support the successful future use of these approaches in combination with others [54].

## 4. The Frequency of PTEN Protein Loss in PDAC Greatly Exceeds That of Reported Changes in the *PTEN* Gene

*PTEN* is one of the most frequently mutated tumour suppressor genes in human cancer [55,56]. Notably, it may provide the greatest selective advantage to tumours carrying mutations compared to other tumour suppressors [57]. In addition to mutations in the *PTEN* gene, numerous other mechanisms have been shown to occur in many cancers that reduce the function of the encoded PTEN phosphatase [55,58,59]. The dominant mechanism by which PTEN functions to oppose tumour formation is as a core inhibitory component of the oncogenic PI3K-AKT signalling network, in which it metabolises the primary lipid products of the class 1 phosphoinositide 3-kinases (PI3Ks) [60,61]. PTEN is a phosphatase, which dephosphorylates position 3 of the inositol ring of PtdInsP_3_ and probably also PtdIns(3,4)P_2_ [59,60,62]. The loss of PTEN function in PDAC is particularly relevant given that KRAS directly binds to and activates the alpha, gamma, and delta isoforms of PI3K [63]. Consequently, PTEN loss is likely to amplify specific downstream signals driven by mutant KRAS.

An important consideration when analysing PTEN loss in tumours is that in some cancer types, PTEN appears to act as a dose-dependent tumour suppressor. Even partial reductions in PTEN protein function can significantly increase cancer risk [64,65]. This mechanism differs from the classical ‘two-hit’ model of recessive tumour suppressor genes, in which only total or near-total loss of function drives cancer.

Genotype–phenotype correlations [66,67] and high-throughput functional assessments of pathogenic PTEN variants provide further support for the hypothesis that a partial loss of PTEN function frequently drives tumour development. These studies indicate that many, if not most, cancer-associated variants retain substantial functional activity [68]. In contrast, for example, almost all cancer-associated mutations in *TP53* show little or no functional activity [69]. Additionally, individuals with Li–Fraumeni and FAMMM syndromes, carrying *TP53* and *CDKN2A* mutations respectively, are commonly found to develop cancers in which the remaining tumour suppressor gene copy is lost [70,71]. Cancers in individuals with PTEN Hamartoma Tumour Syndrome however, rarely display further gene loss [72].

It has been many years since the proposal, based on both human and mouse model data, that haploinsufficient PTEN loss may be a key driver of PDAC in combination with KRAS mutation [73]. Despite this, PTEN and other candidate drivers are often ignored in reviews of PDAC pathogenesis, perhaps because a mutation of the *PTEN* coding sequence is a rare event in pancreatic cancer. For example, at the time of writing (October 2024), the COSMIC database (https://cancer.sanger.ac.uk/cosmic, (accessed on 9 July 2025)) shows 129 mutated samples from 3947 samples analysed (3.27%), and most published studies do not include PTEN in their lists of the most mutated genes in PDAC [7,8,10,11,24]. In cancers affecting other organ systems, the loss of a single copy of PTEN is more commonly observed as the mechanism driving loss of function compared to coding sequence mutations [33,44]. A similar picture is seen in PDAC studies, where all forms of genetic changes in PTEN are uncommon. Reports of PTEN shallow deletions (consistent with hemizygous single copy loss) are generally low (<20%), while deep deletions of PTEN are almost never identified [7,8,11,12,24,74].

Therefore, we subsequently analysed a substantial body of available data that has assessed the abundance of PTEN protein in PDAC tissue samples by immunohistochemistry. We were able to identify ten published research papers containing this data [73,75,76,77,78,79,80,81,82,83], which are summarised in Table 1. As might be expected, these diverse studies use different scoring systems. For example, regarding details of their analysis and scoring of control non-neoplastic pancreatic tissues, Wartenberg and co-workers stated that 13.7% of control pancreatic tissue samples would be scored for ‘PTEN loss’ by the same criteria, which indicate 60% loss for PDAC samples [82]. Another study states that “matched non-neoplastic pancreatic tissue cores retained PTEN expression in all cases examined” [77]. Regardless, across these reports, a consistent picture emerges that PTEN protein is *undetectable* or scored as *negative* in approximately 30% of the PDAC patient samples and scored as relatively *low* or with a *weak* signal in approximately a further 25% of tumours (Table 2). This implies that lost or reduced PTEN function plays a role in driving cancer development in slightly more than half of PDAC cases. Notably, loss of PTEN protein in PDAC samples is associated with a higher tumour grade [76,82], metastasis [76,77,78], and shorter overall survival [75,76,77,81,83].

Researchers have attempted to implement many therapeutic approaches to target proteins and pathways commonly mutated in PDAC, such as KRAS (see Section 3 above) and p53, but this has proved to be challenging, with success in these efforts being currently limited to a small number of patients [86,87]. In contrast, there are a number of FDA-approved drugs that target PI3K, AKT, and mTOR, the three key kinases that make up the growth-promoting signalling network inhibited by PTEN [88,89,90]. Notably, in PDAC, therapeutic inhibition of the PI3K pathway is being explored, not only for its direct effects on cancer cells, but also for its potential benefits on the tumour stroma and anticancer immunity [91].

## 5. Diverse Drivers of PDAC Are Dysregulated at the Protein Level

In addition to genetic alterations, tumour suppressor function can also be affected at the protein level. Several tumour suppressors implicated in PDAC have been studied using immunohistochemistry to assess their protein abundance in tumour samples. The following subsections discuss key proteins whose expression is frequently reduced in PDAC, highlighting their potential roles in tumour development and progression. As described above, genomic studies of PDAC have identified a large number of implicated oncogenes and tumour suppressors in which mutations are identified in tumour samples, many of which do not play major roles in other cancer types. Several of these implicated tumour suppressors have been studied in more detail to investigate the abundance of their encoded proteins in pancreatic cancer samples by immunohistochemistry.

### 5.1. KDM6A/UTX Histone Demethylase

The KDM6A protein is a histone demethylase that also influences gene expression, potentially through the regulation of enhancer function independently of its catalytic activity. Furthermore, loss of KDM6A function has also been implicated in multiple cancers, such as acute myeloid and lymphoblastic leukaemias, myeloma, lung cancer, colorectal cancer, and breast cancer, among others [92,93]. Mutations in the *KDM6A* gene, mostly deletions and truncations, are observed in 3–20% of PDAC samples, and the pancreatic-specific deletion of *Kdm6a* in mice leads to greatly accelerated *Kras*-driven pancreatic cancer [8,13,94,95]. Notably, tumour suppressor activity appears to be largely independent of catalytic activity [92,94]. The expression of the KDM6A protein in PDAC tumour tissue has been investigated by several groups (Table 3), all observing reduced expression of the tumour suppressor in a large proportion of patients’ tumours. The reported fractions of cancers with negative or reduced KDM6A protein in the four studies were 25%, 30%, 69%, and 78% [95,96,97,98]. Together, these data support the conclusion that the reduced expression of KDM6A may contribute to driving tumourigenesis in many, or perhaps most, pancreatic cancers.

### 5.2. ARID1A

The *ARID1A* gene encodes a component of the SWI-SNF chromatin remodelling complex and is subject to loss-of-function mutations in a range of cancer types, including ovarian, endometrial, colorectal, lung, and breast cancers [99,100]. The selective deletion of *Arid1a* in the pancreas of mice results in PanIN formation [101] and accelerates cancer development when combined with the tissue-specific expression of mutant *Kras^K12D^* or with *Pten* deletion [101,102]. Consistent with its role as a tumour suppressor in PDAC, *ARID1A* mutations, which are mostly deletions or truncations, are found in 5–15% of cases of the disease [8,11,13,24]. However, despite this relatively low frequency of genetic alterations, three independent studies of ARID1A protein abundance in pancreatic cancer tissue samples have reported lost or reduced ARID1A expression in 26%, 36%, and 51% of cases [103,104,105]. This is shown in Table 4.

### 5.3. MAP2K4

The *MAP2K4* gene, also known as *MKK4*, encodes a protein kinase that plays a role within Mitogen-Activated Protein Kinase (MAPK) cascades, acting to phosphorylate and activate the MAPK family members JNK1, JNK2, and p38 in response to stresses and mitogens [106]. The selective deletion of *MAP2K4/MKK4* in the pancreas of mice accelerates cancer development when combined with the tissue-specific expression of mutant *Kras^K12D^* [107]. Consistent with the potential to be a tumour suppressor in PDAC, reduced copy number and mutations of *MAP2K4*, including many deletions or truncations, are found in a small fraction of cases. Specific cohort analyses have identified these changes in 2%, 5%, and 24% of patients [8,13,24]. However, immunohistochemical analyses of the abundance of the MAP2K4 protein in pancreatic cancer tissue samples have provided somewhat conflicting data:some studies have reported higher levels of MAP2K4/MKK4 expression in PDAC tissue than in healthy pancreatic ducts, while others have reported a loss of expression [108,109,110,111]. One potential explanation for this discrepancy is a shift in MKK4 protein localisation, as observed in a recent large-scale study, from being predominantly cytoplasmic in healthy tissues to a more nuclear localisation in cancer cells [110]. There is also agreement in larger and more recent studies that loss of the MKK4 protein is associated with a poorer outcome in PDAC cohorts [109,110].

### 5.4. ATM

ATM is a large DNA-damage-activated protein kinase that contributes to the coordination of DNA repair and the maintenance of genome stability [112,113]. It is encoded by the *ATM* gene, in which homozygous loss-of-function germline mutations cause the inherited syndrome Ataxia-Telangiectasia, which is characterised by sensitivity to radiation and elevated cancer risk [113]. Similarly, heterozygous carriers of ATM mutations also have an increased risk of pancreatic cancer, and ATM mutations have been observed in approximately 5% of sporadic cases of PDAC [112]. In accordance with this tumour suppressor status, deletion of *ATM* in mice accelerates RAS-driven pancreatic cancer development and metastasis [114,115]. However, although there is an association of ATM protein loss in PDAC with shorter patient survival, a robust expression of ATM protein is retained in cancer cells in the vast majority (>80%) of PDAC cases [115,116,117]. This uncommon loss of ATM protein expression in PDAC may be related to data showing reduced survival in ATM-negative tumours, which retain the normal p53 protein, but in tumours with aberrant p53 (which comprise the majority of PDACs), ATM status had no effect on survival [116].

When considering the implications of ATM status, this should be viewed as part of the complex relationship between DNA repair and cancer: the loss of genome stability is an important characteristic driving many cancers, but it also makes cancer cells potentially susceptible to genotoxic therapies. This vulnerability is being investigated in multiple cancer types through the development of ATM inhibitors [118] and specifically in PDAC patients with germline BRCA1/2 mutations using the PARP inhibitor Olaparib [119,120], which provides some optimism for future patient outcomes.

### 5.5. GATA6

The GATA6 transcription factor plays an important role in early pancreatic development and cellular differentiation. In the context of PDAC, its expression has been proposed to influence immune infiltration [121]. *GATA6* gene amplification is described in a small subset of PDAC samples (5–20% [8,24,122]), and an increased expression of the GATA6 protein relative to the normal ductal epithelium is reported in approximately half of PDAC cases [122,123]. Notably, high GATA6 expression is associated with the classical PDAC subtype, retained expression of E-Cadherin and CK5, and a longer relative overall survival [121,124,125].

## 6. Conclusions

Many factors affect how specific genetic changes in cancer cells control the function of encoded proteins and cancer development. For example, some tumour suppressor genes (e.g., TP53 and CDKN2A) usually have both gene copies mutated during the development of cancer, whereas others (e.g., PTEN) are more often observed to retain one unmutated gene copy. However, a comprehensive functional proteomic analysis of clinical samples remains far more challenging than an analogous genomic analysis. Therefore, it is easier to accumulate data that provide confidence in the pathogenic role of the former group of drivers than in the latter group, with the risk of overlooking important effects of the latter group.

**Figure 2:**
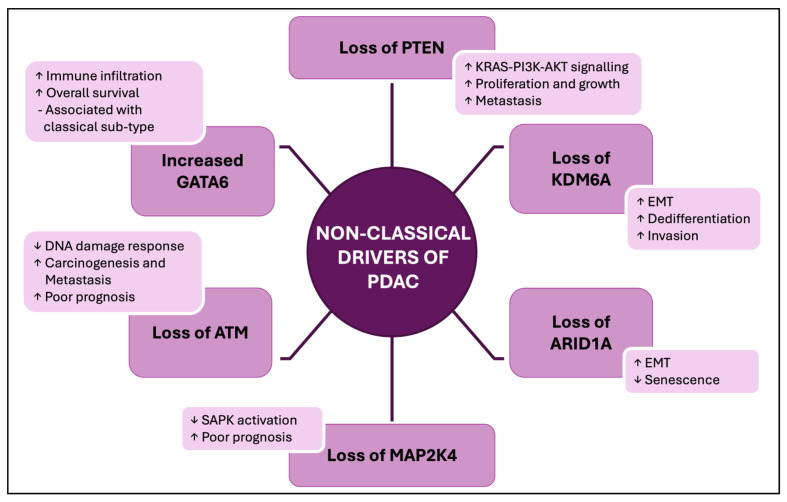
A summary of the changes in tumour suppressor and oncoprotein functions commonly observed in PDAC, which are discussed in this review. Undefined abbreviations: EMT, Epithelial-to-Mesenchymal Transition; SAPK, Stress-Activated Protein Kinase.

We have reviewed data showing reduced levels of a set of tumour suppressor proteins in PDAC tissues observed in a substantial proportion of these cases (with findings summarised in Figure 2).

It is understandable that attention is focused on KRAS, TP53, CDKN2A, and SMAD4, given the evidence for their central roles in the development of pancreatic cancer. Notably, the initial identification of PTEN, KDM6A, and ARID as tumour suppressor proteins was driven by the analysis of mutations in their encoding genes. Furthermore, this reliance on genetics for cancer driver identification is unlikely to change, given the challenges of discovery research to identify tumour suppressors through clinical proteomics.

In this review, we have addressed the non-genomic loss of tumour suppressor function. It should be noted that there are examples of oncogenes that are increased in function in many cases of PDACs in the absence of observed genetic changes. Perhaps most significantly, the increased activation of the Hippo pathway by an increased expression of the Yap1 protein in the absence of mutations appears to be a common (reported in 47% of cases) event in PDAC, in particular in the squamous/basal subtype [126]. The clinical significance of this is supported by data showing that Hippo pathway activation is able to bypass the dependence of PDAC on KRAS mutations and also drive resistance to KRAS G12C inhibitor therapy [127,128].

Regardless, a publication search of the PubMed database with the terms “pancreatic cancer”, “review”, and a selected gene name reveals >150 publications for each of the KRAS, TP53, CDKN2A and SMAD4 genes, but only 16 publications for *PTEN*, 8 for *ARID1A*, and a single review including *KDM6A* within its title or abstract. These analyses, which overlook or underplay other contributors such as PTEN, KDM6A, and ARID1A, may present a limited picture of PDAC pathogenesis. This is particularly true as evidence accumulates for functional links between these proteins [60,98,129,130,131,132,133,134] and highlights the need for future research into the roles of these proteins.

To date, there have only been a small number of studies that have investigated the abundance in PDAC tissues of MAP2K4, ATM, GATA6, and (to a lesser extent) ARID1A and KDM6A proteins. Therefore, while existing findings relating to these proteins appear significant, with few exceptions (e.g., GATA6 as a transcription factor driving early development of the pancreas), there is very limited understanding of why these proteins have specific associations with PDAC rather than other cancer types. Therefore, further data are required to deliver a solid understanding of their role in PDAC and its clinical significance.

PTEN, KDM6A, and ARID1A are not established targets for successful drugs, and their evident loss of function in PDAC does not provide therapeutic strategies that are well-validated in pancreatic cancer patients. However, each of these genes provides potential strategies that are currently under investigation for cancer treatment [135,136] (see Table 5), with some already approved for use in other types of cancer. For instance, the PI3K-AKT-mTOR signalling pathway is directly activated by KRAS and inhibited by PTEN, and several drug classes have been approved that inhibit components of this pathway. For example, inhibitors of mTOR are approved for the treatment of several cancers, including pancreatic neuroendocrine tumours, and they remain under investigation as PDAC therapies, although trials to date have yielded disappointing results [135,137]. Furthermore, inhibitors of class 1 PI3Ks and drugs inhibiting AKT have shown poor results in clinical trials as monotherapies but are now being used in combination therapy for certain groups of breast cancer subtypes. These drugs also remain under investigation in further settings, particularly in cancers displaying PIK3CA mutations or PTEN loss [138,139]. Strategies to target cancers with ARID1A and KDM6A deficiency are not as mature as those targeting the PI3K/AKT/mTOR pathway [99,140]; however, several recent promising results have been reported [97,141,142,143], offering hope for future research.

## Figures and Tables

**Table 1:** Summarised data from studies reporting the percentage of pancreatic cancer samples displaying immunohistochemical loss of SMAD4, p16/INK4A, and p14/ARF protein. The percentage of clinical samples reported as displaying loss of the relevant tumour suppressor protein in each study is shown. The details of scoring methods are found in each publication [25,26,27,28,29,30,31,32].

Study Reference	Xu et al. 2019 [25]	Masugi et al. 2023 [26]	Biankin et al. 2002 [27]	Jeong et al. 2005 [28]	Geradts et al. 2001 [29]	Yu et al. 2004 [30]	Iwatate et al. 2020 [31]	Oshima et al. 2013 [32]
**SMAD4 loss** **(% of samples)**	71	55	53					60
**p16/INK4A loss (% of samples)**	75	85	69	46	100	61	53	67
**p14/ARF loss (% of samples)**					68	65		

**Table 2:** Summarised data from studies reporting the immunohistochemical analysis of PTEN protein levels in human Pancreatic Ductal Adenocarcinoma samples. Different scoring systems were used in different studies, with studies scoring PTEN protein levels either with a digital positive/negative score or using three or four different levels (e.g., high/low/negative). Two studies were identified and excluded from analysis because they used antibodies that are known to give non-specific data in immunohistochemistry [84,85]. Where possible, samples identified as being from pancreatic cancer types other than PDAC were also excluded.

Study Reference	Sample Size	Number of Samples with Stated PTEN Expression			% Patients with Low/Negative Expression
		Positive/High	Low	Negative/<10%	
**Ying et al.** **2011 [73]**	54	16	38		70%
**Boeck et al.** **2013 [75]**	171	141		30	17.5%
**Feng et al.** **2011 [76]**	172	78	94		55%
**Foo et al.** **2013 [77]**	133	99/56	43	34	58%
**Huang et al.** **2016 [78]**	50	19		31	62%
**Jiang et al.** **2014 [79]**	33	7		26	79%
**Pham et al.** **2008 [80]**	26	11	15		58%
**Tao et al.** **2006 [81]**	41	16/7	9	25	61%
**Wartenberg et al. 2016 [82]**	117	47		70	60%
**Zhang et al.** **2020 [83]**	69	11	58		84%

**Table 3:** Summarised data from studies reporting the immunohistochemical analysis of KDM6A protein levels in human PDAC samples. The details of positive/negative or high/low scoring methods are found in each publication [95,96,97,98].

Study Reference	Sample Size	Number of Samples with Stated KDM6A Expression		% Patients with Low/Negative Expression
		Positive/High	Low/Negative/<10%	
**Kalisz et al.** **2020 [95]**	208	46	162	78%
**Watanabe et al. 2018 [96]**	103	77	26	25%
**Yang et al.** **2022 [97]**	13	4	9	69%
**Zhang et al.** **2024 [98]**	60	42	18	30%

**Table 4:** Summarised data from studies reporting the immunohistochemical analysis of ARID1A protein levels in human Pancreatic Ductal Adenocarcinoma samples. The details of positive/negative or high/low scoring methods are found in each publication [103,104,105].

Study Reference	Sample Size	Number of Samples with stated ARID1A Expression		% Patients with Low/Negative Expression
		Positive/High	Low/Negative/<10%	
**Numata et al. 2013 [103]**	39	19	20	51%
**Zhang et al. 2018 [104]**	73	54	19	26%
**Kimura et al. 2018 [105]**	44	28	16	36%

**Table 5:** Selected ongoing clinical trials including PDAC patients, and with relevance to the proteins discussed in the review. Further details of each trial are available at clinicaltrials.gov. Additionally, there are approximately ten trials underway investigating the selective inhibitors of KRAS G12C that vary with regard to the drug and drug combinations, which are not listed.

Trial	Phase	Therapeutic Drug(s)	Target(s)	Details
**NCT06078800**	1	YL-17231	**KRAS**	Non-mutation-specific inhibitor
**NCT06607185**	1	LY4066434	**KRAS**	Non-mutation-specific inhibitor. Estimated 750 participants
**NCT05057013**	1/2a	HMBD001	**HER3**	MAb
**NCT03065062**	1	Palbociclib and Gedatolisib	**CDK4/6** **PI3K/mTOR**	
**NCT02433626**	1	COTI-2	**p53 and PI3K**	Re-activator of mutant p53 also activates PI3K signalling
**NCT03682289**	2	Ceralasertib	**ATR**	Combined with PARP and PDL1 inhibitors. Informed by ARID1A IHC status
**NCT05068752**	2	Vemurafenib	**BRAF**	Combined with Sorafenib multikinase inhibitor
**NCT06770452**	2	HRS-4642	**KRAS G12D**	Patients with KRAS G12D mutation. Combined with EGFR MAb Nimotuzumab
**NCT07026916**	2	GFH375	**KRAS G12D**	Patients with KRAS G12D mutation
**NCT06998940**	3	Panitumumab	**EGFR**	PDAC without KRAS mutation

## References

[1] Siegel R.L., Miller K.D., Wagle N.S., Jemal A. (2023). Cancer Statistics, 2023. CA A Cancer J. Clin..

[2] Ferlay J., Partensky C., Bray F. (2016). More Deaths from Pancreatic Cancer Than Breast Cancer in the EU by 2017. Acta Oncol..

[3] Kleeff J., Korc M., Apte M., La Vecchia C., Johnson C.D., Biankin A.V., Neale R.E., Tempero M., Tuveson D.A., Hruban R.H. (2016). Pancreatic Cancer. Nat. Rev. Dis. Primers.

[4] Karamitopoulou E. (2019). Tumour Microenvironment of Pancreatic Cancer: Immune landscape is Dictated by Molecular and Histopathological Features. Br. J. Cancer.

[5] Erkan M., Hausmann S., Michalski C.W., Fingerle A.A., Dobritz M., Kleeff J., Friess H. (2012). The Role of Stroma in Pancreatic Cancer: Diagnostic and Therapeutic Implications. Nat. Rev. Gastroenterol. Hepatol..

[6] Notta F., Hahn S.A., Real F.X. (2017). A Genetic Roadmap of Pancreatic Cancer: Still Evolving. Gut.

[7] Biankin A.V., Waddell N., Kassahn K.S., Gingras M.C., Muthuswamy L.B., Johns A.L., Miller D.K., Wilson P.J., Patch A.M., Wu J. (2012). Pancreatic Cancer Genomes Reveal Aberrations in Axon Guidance Pathway Genes. Nature.

[8] Waddell N., Pajic M., Patch A.M., Chang D.K., Kassahn K.S., Bailey P., Johns A.L., Miller D., Nones K., Quek K. (2015). Whole Genomes Redefine the Mutational Landscape of Pancreatic Cancer. Nature.

[9] Ying H., Dey P., Yao W., Kimmelman A.C., Draetta G.F., Maitra A., DePinho R.A. (2016). Genetics and Biology of Pancreatic Ductal Adenocarcinoma. Genes Dev..

[10] Jones S., Zhang X., Parsons D.W., Lin J.C., Leary R.J., Angenendt P., Mankoo P., Carter H., Kamiyama H., Jimeno A. (2008). Core Signaling Pathways in Human Pancreatic Cancers Revealed by Global Genomic Analyses. Science.

[11] Lu J., Yu R., Liu R., Liang X., Sun J., Zhang H., Wu H., Zhang Z., Shao Y.W., Guo J. (2021). Genetic Aberrations in Chinese Pancreatic Cancer Patients and Their Association with Anatomic Location and Disease Outcomes. Cancer Med..

[12] Takano S., Fukasawa M., Shindo H., Takahashi E., Hirose S., Fukasawa Y., Kawakami S., Hayakawa H., Kuratomi N., Kadokura M. (2021). Clinical Significance of Genetic Alterations in Endoscopically Obtained Pancreatic Cancer Specimens. Cancer Med..

[13] Bailey P., Chang D.K., Nones K., Johns A.L., Patch A.M., Gingras M.C., Miller D.K., Christ A.N., Bruxner T.J., Quinn M.C. (2016). Genomic Analyses Identify Molecular Subtypes of Pancreatic Cancer. Nature.

[14] Biankin A.V., Maitra A. (2015). Subtyping Pancreatic Cancer. Cancer Cell.

[15] Sausen M., Phallen J., Adleff V., Jones S., Leary R.J., Barrett M.T., Anagnostou V., Parpart-Li S., Murphy D., Kay Li Q. (2015). Clinical Implications of Genomic Alterations in the Tumour and Circulation of Pancreatic Cancer Patients. Nat. Commun..

[16] Kanda M., Matthaei H., Wu J., Hong S.M., Yu J., Borges M., Hruban R.H., Maitra A., Kinzler K., Vogelstein B. (2012). Presence of Somatic Mutations in Most Early-Stage Pancreatic Intraepithelial Neoplasia. Gastroenterology.

[17] Vincent A., Herman J., Schulick R., Hruban R.H., Goggins M. (2011). Pancreatic cancer. Lancet.

[18] Connor A.A., Gallinger S. (2022). Pancreatic Cancer Evolution and Heterogeneity: Integrating Omics and Clinical Data. Nat. Rev. Cancer.

[19] Buday L., Downward J. (2008). Many Faces of Ras Activation. Biochim. Biophys. Acta.

[20] Uprety D., Adjei A.A. (2020). KRAS: From Undruggable to a Druggable Cancer Target. Cancer Treat. Rev..

[21] Liu Y., Su Z., Tavana O., Gu W. (2024). Understanding the Complexity of p53 in a New Era of Tumor Suppression. Cancer Cell.

[22] Kim W.Y., Sharpless N.E. (2006). The Regulation of INK4/ARF in Cancer and Aging. Cell.

[23] McCarthy A.J., Chetty R. (2018). Smad4/DPC4. J. Clin. Pathol..

[24] Singhi A.D., George B., Greenbowe J.R., Chung J., Suh J., Maitra A., Klempner S.J., Hendifar A., Milind J.M., Golan T. (2019). Real-Time Targeted Genome Profile Analysis of Pancreatic Ductal Adenocarcinomas Identifies Genetic Alterations That Might Be Targeted With Existing Drugs or Used as Biomarkers. Gastroenterology.

[25] Xu J.Z., Wang W.Q., Zhang W.H., Xu H.X., Gao H.L., Zhang S.R., Wu C.T., Li S., Li H., Xu J. (2019). The Loss of SMAD4/DPC4 Expression Associated with a Strongly Activated Hedgehog Signaling Pathway Predicts Poor Prognosis in Resected Pancreatic Cancer. J. Cancer.

[26] Masugi Y., Takamatsu M., Tanaka M., Hara K., Inoue Y., Hamada T., Suzuki T., Arita J., Hirose Y., Kawaguchi Y. (2023). Post-Operative Mortality and Recurrence Patterns in Pancreatic Cancer According to KRAS Mutation and CDKN2A, p53, and SMAD4 Expression. J. Pathol. Clin. Res..

[27] Biankin A.V., Morey A.L., Lee C.S., Kench J.G., Biankin S.A., Hook H.C., Head D.R., Hugh T.B., Sutherland R.L., Henshall S.M. (2002). DPC4/Smad4 Expression and Outcome in Pancreatic Ductal Adenocarcinoma. J. Clin. Oncol..

[28] Jeong J., Park Y.N., Park J.S., Yoon D.S., Chi H.S., Kim B.R. (2005). Clinical Significance of P16 Protein Expression Loss and Aberrant P53 Protein Expression in Pancreatic Cancer. Yonsei Med. J..

[29] Geradts J., Wilentz R.E., Roberts H. (2001). Immunohistochemical [Corrected] Detection of the Alternate INK4a-Encoded Tumor Suppressor Protein p14(ARF) in Archival Human Cancers and Cell Lines Using Commercial Antibodies: Correlation with P16(INK4a) Expression. Mod. Pathol..

[30] Yu G.Z., Zhu M.H., Ni C.R., Li F.M., Zheng J.M., Gong Z.J. (2004). Expression of Proteins in P53 (P14arf-Mdm2-P53-P21waf/Cip1) Pathway and Their Significance in Exocrine Pancreatic Carcinoma. Zhonghua Bing Li Xue Za Zhi.

[31] Iwatate Y., Hoshino I., Ishige F., Itami M., Chiba S., Arimitsu H., Yanagibashi H., Nagase H., Yokota H., Takayama W. (2020). Prognostic Significance of P16 Protein in Pancreatic Ductal Adenocarcinoma. Mol. Clin. Oncol..

[32] Oshima M., Okano K., Muraki S., Haba R., Maeba T., Suzuki Y., Yachida S. (2013). Immunohistochemically Detected Expression of 3 Major Genes (CDKN2A/p16, TP53, and SMAD4/DPC4) Strongly Predicts Survival in Patients with Resectable Pancreatic Cancer. Ann. Surg..

[33] Buckley C.W., O’Reilly E.M. (2024). Next-Generation Therapies for Pancreatic Cancer. Expert Rev. Gastroenterol. Hepatol..

[34] Golan T., Hammel P., Reni M., Van Cutsem E., Macarulla T., Hall M.J., Park J.O., Hochhauser D., Arnold D., Oh D.Y. (2019). Maintenance Olaparib for Germline BRCA-Mutated Metastatic Pancreatic Cancer. N. Engl. J. Med..

[35] Vaishampayan U.N. (2021). An Evaluation of Olaparib for the Treatment of Pancreatic Cancer. Expert Opin. Pharmacother..

[36] M’Baloula J., Tougeron D., Boileve A., Jeanbert E., Guimbaud R., Ben Abdelghani M., Durand A., Turpin A., Quesada S., Blanc J.F. (2024). Olaparib as Maintenance Therapy in Non Resectable Pancreatic Adenocarcinoma Associated with Homologous Recombination Deficiency Profile: A French Retrospective Multicentric AGEO Real-World Study. Eur. J. Cancer.

[37] Mehra T., Lupatsch J.E., Kossler T., Dedes K., Siebenhuner A.R., von Moos R., Wicki A., Schwenkglenks M.E. (2024). Olaparib Not Cost-Effective as Maintenance Therapy for Platinum-Sensitive, BRCA1/2 Germline-Mutated Metastatic Pancreatic Cancer. PLoS ONE.

[38] Hong D.S., Fakih M.G., Strickler J.H., Desai J., Durm G.A., Shapiro G.I., Falchook G.S., Price T.J., Sacher A., Denlinger C.S. (2020). KRAS(G12C) Inhibition with Sotorasib in Advanced Solid Tumors. N. Engl. J. Med..

[39] Skoulidis F., Li B.T., Dy G.K., Price T.J., Falchook G.S., Wolf J., Italiano A., Schuler M., Borghaei H., Barlesi F. (2021). Sotorasib for Lung Cancers with KRAS p.G12C Mutation. N. Engl. J. Med..

[40] Ostrem J.M., Peters U., Sos M.L., Wells J.A., Shokat K.M. (2013). K-Ras(G12C) Inhibitors Allosterically Control GTP Affinity and Effector Interactions. Nature.

[41] Zhou C.C., Li C.Y., Luo L.B., Li X., Jia K.Y., He N., Mao S.Q., Wang W.Y., Shao C.C., Liu X.Y. (2024). Anti-tumor Efficacy of HRS-4642 and Its Potential Combination with Proteasome Inhibition in KRAS-Mutant Cancer. Cancer Cell.

[42] Li Y., Zhao J.F., Li Y.T. (2025). New exploration of KRAS Inhibitors and the Mechanisms of Resistance. Exp. Hematol. Oncol..

[43] Cancer Genome Atlas Research Network (2017). Integrated Genomic Characterization of Pancreatic Ductal Adenocarcinoma. Cancer Cell.

[44] Collisson E.A., Sadanandam A., Olson P., Gibb W.J., Truitt M., Gu S., Cooc J., Weinkle J., Kim G.E., Jakkula L. (2011). Subtypes of Pancreatic Ductal Adenocarcinoma and Their Differing Responses to Therapy. Nat. Med..

[45] Moffitt R.A., Marayati R., Flate E.L., Volmar K.E., Loeza S.G., Hoadley K.A., Rashid N.U., Williams L.A., Eaton S.C., Chung A.H. (2015). Virtual Microdissection Identifies Distinct Tumor- and Stroma-Specific Subtypes of Pancreatic Ductal Adenocarcinoma. Nat. Genet..

[46] Huang X., Zhang G., Liang T. (2022). Subtyping for Pancreatic Cancer Precision Therapy. Trends Pharmacol. Sci..

[47] Ruff S.M., Pawlik T.M. (2024). Molecular Classification and Pathogenesis of Pancreatic Adenocarcinoma and Targeted Therapies: A Review. Front. Biosci. (Landmark Ed).

[48] Waldman A.D., Fritz J.M., Lenardo M.J. (2020). A Guide to Cancer Immunotherapy: From T Cell Basic Science to Clinical Practice. Nat. Rev. Immunol..

[49] Ho W.J., Jaffee E.M., Zheng L. (2020). The Tumour Microenvironment in Pancreatic Cancer—Clinical Challenges and Opportunities. Nat. Rev. Clin. Oncol..

[50] Jiang H., Torphy R.J., Steiger K., Hongo H., Ritchie A.J., Kriegsmann M., Horst D., Umetsu S.E., Joseph N.M., McGregor K. (2020). Pancreatic Ductal Adenocarcinoma Progression is Restrained by Stromal Matrix. J. Clin. Investig..

[51] Jiang H., Liu X., Knolhoff B.L., Hegde S., Lee K.B., Jiang H., Fields R.C., Pachter J.A., Lim K.H., DeNardo D.G. (2020). Development of Resistance to FAK Inhibition in Pancreatic Cancer is Linked to Stromal Depletion. Gut.

[52] Grunwald B.T., Devisme A., Andrieux G., Vyas F., Aliar K., McCloskey C.W., Macklin A., Jang G.H., Denroche R., Romero J.M. (2021). Spatially Confined Sub-Tumor Microenvironments in Pancreatic Cancer. Cell.

[53] Raghavan S., Winter P.S., Navia A.W., Williams H.L., DenAdel A., Lowder K.E., Galvez-Reyes J., Kalekar R.L., Mulugeta N., Kapner K.S. (2021). Microenvironment Drives Cell State, Plasticity, and Drug Response in Pancreatic Cancer. Cell.

[54] Liu T., Cheng S., Xu Q., Wang Z. (2022). Management of Advanced Pancreatic Cancer through Stromal Depletion and Immune Modulation. Medicina.

[55] Alvarez-Garcia V., Tawil Y., Wise H.M., Leslie N.R. (2019). Mechanisms of PTEN Loss in Cancer: It’s All About Diversity. Semin. Cancer Biol..

[56] Worby C.A., Dixon J.E. (2014). Pten. Annu. Rev. Biochem..

[57] Bignell G.R., Greenman C.D., Davies H., Butler A.P., Edkins S., Andrews J.M., Buck G., Chen L., Beare D., Latimer C. (2010). Signatures of Mutation and Selection in the Cancer Genome. Nature.

[58] Leslie N.R., Foti M. (2011). Non-Genomic Loss of PTEN Function in Cancer: Not in My Genes. Trends Pharmacol. Sci..

[59] Song M.S., Salmena L., Pandolfi P.P. (2012). The Functions and Regulation of the PTEN Tumour Suppressor. Nat. Rev. Mol. Cell Biol..

[60] Vanhaesebroeck B., Stephens L., Hawkins P. (2012). PI3K signalling: The Path to Discovery and Understanding. Nat. Rev. Mol. Cell Biol..

[61] Fruman D.A., Chiu H., Hopkins B.D., Bagrodia S., Cantley L.C., Abraham R.T. (2017). The PI3K Pathway in Human Disease. Cell.

[62] Malek M., Kielkowska A., Chessa T., Anderson K.E., Barneda D., Pir P., Nakanishi H., Eguchi S., Koizumi A., Sasaki J. (2017). PTEN Regulates PI(3,4)P(2) Signaling Downstream of Class I PI3K. Mol. Cell.

[63] Fritsch R., de Krijger I., Fritsch K., George R., Reason B., Kumar M.S., Diefenbacher M., Stamp G., Downward J. (2013). RAS and RHO Families of GTPases Directly Regulate Distinct Phosphoinositide 3-Kinase Isoforms. Cell.

[64] Alimonti A., Carracedo A., Clohessy J.G., Trotman L.C., Nardella C., Egia A., Salmena L., Sampieri K., Haveman W.J., Brogi E. (2010). Subtle Variations in Pten Dose Determine Cancer Susceptibility. Nat. Genet..

[65] Berger A.H., Knudson A.G., Pandolfi P.P. (2011). A Continuum Model for Tumour Suppression. Nature.

[66] Rodriguez-Escudero I., Oliver M.D., Andres-Pons A., Molina M., Cid V.J., Pulido R. (2011). A comprehensive functional analysis of PTEN mutations: Implications in Tumor- and Autism-Related Syndromes. Hum. Mol. Genet..

[67] Spinelli L., Black F.M., Berg J.N., Eickholt B.J., Leslie N.R. (2015). Functionally Distinct Groups of Inherited PTEN Mutations in Autism and Tumour Syndromes. J. Med. Genet..

[68] Mighell T.L., Evans-Dutson S., O’Roak B.J. (2018). A Saturation Mutagenesis Approach to Understanding PTEN Lipid Phosphatase Activity and Genotype-Phenotype Relationships. Am. J. Hum. Genet..

[69] Carbonnier V., Leroy B., Rosenberg S., Soussi T. (2020). Comprehensive Assessment of TP53 Loss of Function Using Multiple Combinatorial Mutagenesis Libraries. Sci. Rep..

[70] Christodoulou E., Nell R.J., Verdijk R.M., Gruis N.A., van der Velden P.A., van Doorn R. (2020). Loss of Wild-Type CDKN2A Is an Early Event in the Development of Melanoma in FAMMM Syndrome. J. Investig. Dermatol..

[71] Shetzer Y., Kagan S., Koifman G., Sarig R., Kogan-Sakin I., Charni M., Kaufman T., Zapatka M., Molchadsky A., Rivlin N. (2014). The Onset of P53 Loss of Heterozygosity is Differentially Induced in Various Stem Cell Types and May Involve the loss of Either Allele. Cell Death Differ..

[72] Leslie N.R., Longy M. (2016). Inherited PTEN Mutations and the Prediction of Phenotype. Semin. Cell Dev. Biol..

[73] Ying H., Elpek K.G., Vinjamoori A., Zimmerman S.M., Chu G.C., Yan H., Fletcher-Sananikone E., Zhang H., Liu Y., Wang W. (2011). PTEN is a Major Tumor Suppressor in Pancreatic Ductal Adenocarcinoma and Regulates an NF-Kappab-Cytokine Network. Cancer Discov..

[74] Vidotto T., Melo C.M., Lautert-Dutra W., Chaves L.P., Reis R.B., Squire J.A. (2023). Pan-Cancer Genomic Analysis Shows Hemizygous PTEN Loss Tumors are Associated with Immune Evasion and Poor Outcome. Sci. Rep..

[75] Boeck S., Jung A., Laubender R.P., Neumann J., Egg R., Goritschan C., Vehling-Kaiser U., Winkelmann C., Fischer von Weikersthal L., Clemens M.R. (2013). EGFR Pathway Biomarkers in Erlotinib-Treated Patients with Advanced Pancreatic Cancer: Translational Results from the Randomised, Crossover Phase 3 Trial AIO-PK0104. Br. J. Cancer.

[76] Feng C., Yao R., Huang F., Liu X., Nie W. (2011). High Level of PTEN Expression is Associated with Low-Grade Liver Metastasis and Satisfactory Patient Survival in Pancreatic Cancer. Arch. Med. Res..

[77] Foo W.C., Rashid A., Wang H., Katz M.H., Lee J.E., Pisters P.W., Wolff R.A., Abbruzzese J.L., Fleming J.B., Wang H. (2013). Loss of Phosphatase and Tensin Homolog Expression is Associated with Recurrence and Poor Prognosis in Patients with Pancreatic Ductal Adenocarcinoma. Hum. Pathol..

[78] Huang W., Yang J., Ren J., Tang J. (2016). Expression of PTEN and KAI1 Tumor Suppressor Genes in Pancreatic Carcinoma and its Association with Different Pathological Factors. Oncol. Lett..

[79] Jiang K., Lawson D., Cohen C., Siddiqui M.T. (2014). Galectin-3 and PTEN Expression in Pancreatic Ductal Adenocarcinoma, Pancreatic Neuroendocrine Neoplasms and Gastrointestinal Tumors on Fine-Needle Aspiration Cytology. Acta Cytol..

[80] Pham N.A., Schwock J., Iakovlev V., Pond G., Hedley D.W., Tsao M.S. (2008). Immunohistochemical Analysis of Changes in Signaling Pathway Activation Downstream of Growth Factor Receptors in Pancreatic Duct Cell Carcinogenesis. BMC Cancer.

[81] Tao J., Xiong J., Li T., Yang Z., Li X., Li K., Wu H., Wang C. (2006). Correlation Between Protein Expression of PTEN in Human Pancreatic Cancer and the Proliferation, Infiltration, Metastasis and Prognosis. J. Huazhong Univ. Sci. Technolog. Med. Sci..

[82] Wartenberg M., Centeno I., Haemmig S., Vassella E., Zlobec I., Galvan J.A., Neuenschwander M., Schlup C., Gloor B., Lugli A. (2016). PTEN Alterations of the Stromal Cells Characterise an Aggressive Subpopulation of Pancreatic Cancer with Enhanced Metastatic Potential. Eur. J. Cancer..

[83] Zhang Q., Li X., Li Y., Chen S., Shen X., Dong X., Song Y., Zhang X., Huang K. (2020). Expression of the PTEN/FOXO3a/PLZF Signalling Pathway in Pancreatic Cancer and its Significance in Tumourigenesis and Progression. Investig. New Drugs.

[84] Leslie N.R., Yang X., Downes C.P., Weijer C.J. (2007). PtdIns(3,4,5)P3-Dependent and -Independent Roles for PTEN in the Control of Cell Migration. Curr. Biol..

[85] Pallares J., Bussaglia E., Martinez-Guitarte J.L., Dolcet X., Llobet D., Rue M., Sanchez-Verde L., Palacios J., Prat J., Matias-Guiu X. (2005). Immunohistochemical Analysis of PTEN in Endometrial Carcinoma: A Tissue Microarray Study with a Comparison of Four Commercial Antibodies in Correlation with Molecular Abnormalities. Mod. Pathol..

[86] Hassin O., Oren M. (2023). Drugging P53 in Cancer: One Protein, Many Targets. Nat. Rev. Drug Discov..

[87] Moore A.R., Rosenberg S.C., McCormick F., Malek S. (2020). RAS-Targeted Therapies: Is the Undruggable Drugged?. Nat. Rev. Drug Discov..

[88] Turner N.C., Oliveira M., Howell S.J., Dalenc F., Cortes J., Gomez Moreno H.L., Hu X., Jhaveri K., Krivorotko P., Loibl S. (2023). Capivasertib in Hormone Receptor-Positive Advanced Breast Cancer. N. Engl. J. Med..

[89] De Santis M.C., Gulluni F., Campa C.C., Martini M., Hirsch E. (2019). Targeting PI3K Signaling in Cancer: Challenges and Advances. Biochim. Biophys. Acta Rev. Cancer.

[90] Yu L., Wei J., Liu P. (2022). Attacking the PI3K/Akt/mTOR Signaling Pathway for Targeted Therapeutic Treatment in Human Cancer. Semin. Cancer Biol..

[91] Sun P., Meng L.H. (2020). Emerging Roles of Class I PI3K Inhibitors in Modulating Tumor Microenvironment and Immunity. Acta Pharmacol. Sin..

[92] Tran N., Broun A., Ge K. (2020). Lysine Demethylase KDM6A in Differentiation, Development, and Cancer. Mol. Cell. Biol..

[93] van Haaften G., Dalgliesh G.L., Davies H., Chen L., Bignell G., Greenman C., Edkins S., Hardy C., O’Meara S., Teague J. (2009). Somatic Mutations of the Histone H3K27 Demethylase Gene UTX in Human Cancer. Nat. Genet..

[94] Andricovich J., Perkail S., Kai Y., Casasanta N., Peng W., Tzatsos A. (2018). Loss of KDM6A Activates Super-Enhancers to Induce Gender-Specific Squamous-like Pancreatic Cancer and Confers Sensitivity to BET Inhibitors. Cancer Cell.

[95] Kalisz M., Bernardo E., Beucher A., Maestro M.A., Del Pozo N., Millan I., Haeberle L., Schlensog M., Safi S.A., Knoefel W.T. (2020). HNF1A Recruits KDM6A to Activate Differentiated Acinar Cell Programs that Suppress Pancreatic Cancer. EMBO J..

[96] Watanabe S., Shimada S., Akiyama Y., Ishikawa Y., Ogura T., Ogawa K., Ono H., Mitsunori Y., Ban D., Kudo A. (2019). Loss of KDM6A Characterizes a Poor Prognostic Subtype of Human Pancreatic Cancer and Potentiates HDAC Inhibitor Lethality. Int. J. Cancer.

[97] Yang J., Jin L., Kim H.S., Tian F., Yi Z., Bedi K., Ljungman M., Pasca di Magliano M., Crawford H., Shi J. (2022). KDM6A Loss Recruits Tumor-Associated Neutrophils and Promotes Neutrophil Extracellular Trap Formation in Pancreatic Cancer. Cancer Res..

[98] Zhang H.Q., Kong F., Kong X., Jiang T., Ma M., Zheng S., Guo J., Xie K. (2024). Loss of GATA6-Mediated Up-Regulation of UTX Promotes Pancreatic Tumorigenesis and Progression. Genes Dis..

[99] Mullen J., Kato S., Sicklick J.K., Kurzrock R. (2021). Targeting ARID1A Mutations in Cancer. Cancer Treat. Rev..

[100] Wu J.N., Roberts C.W. (2013). ARID1A Mutations in Cancer: Another Epigenetic Tumor Suppressor?. Cancer Discov..

[101] Wang S.C., Nassour I., Xiao S., Zhang S., Luo X., Lee J., Li L., Sun X., Nguyen L.H., Chuang J.C. (2019). SWI/SNF Component ARID1A Restrains Pancreatic Neoplasia Formation. Gut.

[102] Fukunaga Y., Fukuda A., Omatsu M., Namikawa M., Sono M., Masuda T., Araki O., Nagao M., Yoshikawa T., Ogawa S. (2022). Loss of Arid1a and Pten in Pancreatic Ductal Cells Induces Intraductal Tubulopapillary Neoplasm via the YAP/TAZ Pathway. Gastroenterology.

[103] Numata M., Morinaga S., Watanabe T., Tamagawa H., Yamamoto N., Shiozawa M., Nakamura Y., Kameda Y., Okawa S., Rino Y. (2013). The Clinical Significance of SWI/SNF Complex in Pancreatic Cancer. Int. J. Oncol..

[104] Zhang L., Wang C., Yu S., Jia C., Yan J., Lu Z., Chen J. (2018). Loss of ARID1A Expression Correlates With Tumor Differentiation and Tumor Progression Stage in Pancreatic Ductal Adenocarcinoma. Technol. Cancer Res. Treat..

[105] Kimura Y., Fukuda A., Ogawa S., Maruno T., Takada Y., Tsuda M., Hiramatsu Y., Araki O., Nagao M., Yoshikawa T. (2018). ARID1A Maintains Differentiation of Pancreatic Ductal Cells and Inhibits Development of Pancreatic Ductal Adenocarcinoma in Mice. Gastroenterology.

[106] Haeusgen W., Herdegen T., Waetzig V. (2011). The bottleneck of JNK signaling: Molecular and Functional Characteristics of MKK4 and MKK7. Eur. J. Cell Biol..

[107] Davies C.C., Harvey E., McMahon R.F., Finegan K.G., Connor F., Davis R.J., Tuveson D.A., Tournier C. (2014). Impaired JNK Signaling Cooperates with KrasG12D Expression to Accelerate Pancreatic Ductal Adenocarcinoma. Cancer Res..

[108] Handra-Luca A., Lesty C., Hammel P., Sauvanet A., Rebours V., Martin A., Fagard R., Flejou J.F., Faivre S., Bedossa P. (2012). Biological and Prognostic Relevance of Mitogen-Activated Protein Kinases in Pancreatic Adenocarcinoma. Pancreas.

[109] Liu X.D., Zhang Z.W., Wu H.W., Liang Z.Y. (2021). A New Prognosis Prediction Model Combining TNM stage with MAP2K4 and JNK in Postoperative Pancreatic Cancer Patients. Pathol. Res. Pract..

[110] Lu J., Zhou L., Yang G., Liang Z.Y., Zhou W.X., You L., Yuan D., Li B.Q., Guo J.C., Zhao Y.P. (2019). Clinicopathological and Prognostic Significance of MKK4 and MKK7 in Resectable Pancreatic Ductal Adenocarcinoma. Hum. Pathol..

[111] Xin W., Yun K.J., Ricci F., Zahurak M., Qiu W., Su G.H., Yeo C.J., Hruban R.H., Kern S.E., Iacobuzio-Donahue C.A. (2004). MAP2K4/MKK4 expression in Pancreatic Cancer: Genetic Validation of Immunohistochemistry and Relationship to Disease Course. Clin. Cancer Res..

[112] Armstrong S.A., Schultz C.W., Azimi-Sadjadi A., Brody J.R., Pishvaian M.J. (2019). ATM Dysfunction in Pancreatic Adenocarcinoma and Associated Therapeutic Implications. Mol. Cancer Ther..

[113] Lee J.H., Paull T.T. (2021). Cellular Functions of the Protein Kinase ATM and Their Relevance to Human Disease. Nat. Rev. Mol. Cell Biol..

[114] Drosos Y., Escobar D., Chiang M.Y., Roys K., Valentine V., Valentine M.B., Rehg J.E., Sahai V., Begley L.A., Ye J. (2017). ATM-Deficiency Increases Genomic Instability and Metastatic Potential in a Mouse Model of Pancreatic Cancer. Sci. Rep..

[115] Russell R., Perkhofer L., Liebau S., Lin Q., Lechel A., Feld F.M., Hessmann E., Gaedcke J., Guthle M., Zenke M. (2015). Loss of ATM Accelerates Pancreatic Cancer Formation and Epithelial-Mesenchymal Transition. Nat. Commun..

[116] Kim H., Saka B., Knight S., Borges M., Childs E., Klein A., Wolfgang C., Herman J., Adsay V.N., Hruban R.H. (2014). Having Pancreatic Cancer with Tumoral Loss of ATM and Normal TP53 Protein Expression is Associated with a Poorer Prognosis. Clin. Cancer Res..

[117] Paranal R.M., Jiang Z., Hutchings D., Kryklyva V., Gauthier C., Fujikura K., Nanda N., Huang B., Skaro M., Wolfgang C.L. (2023). Somatic Loss of ATM is a Late Event in Pancreatic Tumorigenesis. J. Pathol..

[118] Du S., Liang Q., Shi J. (2024). Progress of ATM Inhibitors: Opportunities and Challenges. Eur. J. Med. Chem..

[119] Perkhofer L., Gout J., Roger E., Kude de Almeida F., Baptista Simoes C., Wiesmuller L., Seufferlein T., Kleger A. (2021). DNA Damage Repair as a Target in Pancreatic Cancer: State-of-the-art and Future Perspectives. Gut.

[120] Perkhofer L., Schmitt A., Romero Carrasco M.C., Ihle M., Hampp S., Ruess D.A., Hessmann E., Russell R., Lechel A., Azoitei N. (2017). ATM Deficiency Generating Genomic Instability Sensitizes Pancreatic Ductal Adenocarcinoma Cells to Therapy-Induced DNA Damage. Cancer Res..

[121] van Eijck C.W.F., Real F.X., Malats N., Vadgama D., van den Bosch T.P.P., Doukas M., van Eijck C.H.J., Mustafa D.A.M., Dutch Pancreatic Cancer G. (2024). GATA6 Identifies An Immune-Enriched Phenotype Linked to Favorable Outcomes in Patients with Pancreatic Cancer Undergoing Upfront Surgery. Cell Rep. Med..

[122] Kwei K.A., Bashyam M.D., Kao J., Ratheesh R., Reddy E.C., Kim Y.H., Montgomery K., Giacomini C.P., Choi Y.L., Chatterjee S. (2008). Genomic Profiling Identifies GATA6 as a Candidate Oncogene Amplified In Pancreatobiliary Cancer. PLoS Genet..

[123] Fu B., Luo M., Lakkur S., Lucito R., Iacobuzio-Donahue C.A. (2008). Frequent Genomic Copy Number Gain and Overexpression of GATA-6 in Pancreatic Carcinoma. Cancer Biol. Ther..

[124] Kokumai T., Omori Y., Ishida M., Ohtsuka H., Mizuma M., Nakagawa K., Maeda C., Ono Y., Mizukami Y., Miura S. (2023). GATA6 and CK5 Stratify the Survival of Patients With Pancreatic Cancer Undergoing Neoadjuvant Chemotherapy. Mod. Pathol..

[125] Shibayama T., Hayashi A., Toki M., Kitahama K., Ho Y.J., Kato K., Yamada T., Kawamoto S., Kambayashi K., Ochiai K. (2024). Combination Immunohistochemistry for CK5/6, p63, GATA6, and HNF4a Predicts Clinical Outcome In Treatment-Naive Pancreatic Ductal Adenocarcinoma. Sci. Rep..

[126] Tu B., Yao J., Ferri-Borgogno S., Zhao J., Chen S.J., Wang Q.Y., Yan L., Zhou X., Zhu C.H., Bang S.M. (2019). YAP1 Oncogene is a Context-Specific Driver for Pancreatic Ductal Adenocarcinoma. JCI Insight.

[127] Kapoor A., Yao W.T., Ying H.Q., Hua S.J., Liewen A., Wang Q.Y., Zhong Y., Wu C.J., Sadanandam A., Hu B.L. (2014). Yap1 Activation Enables Bypass of Oncogenic Kras Addiction in Pancreatic Cancer. Cell.

[128] Mukhopadhyay S., Huang H.Y., Lin Z.Y., Ranieri M., Li S., Sahu S., Liu Y.Z., Ban Y., Guidry K., Hu H. (2023). Genome-Wide CRISPR Screens Identify Multiple Synthetic Lethal Targets That Enhance KRAS Inhibitor Efficacy. Cancer Res..

[129] Tamagawa H., Fujii M., Togasaki K., Seino T., Kawasaki S., Takano A., Toshimitsu K., Takahashi S., Ohta Y., Matano M. (2024). Wnt-deficient and Hypoxic Environment Orchestrates Squamous Reprogramming of human Pancreatic Ductal Adenocarcinoma. Nat. Cell Biol..

[130] Yao H., Luo L., Li R., Zhao Y., Zhang L., Pesic M., Cai L., Li L. (2024). New Insight into the Role of SMAD4 Mutation/Deficiency in the Prognosis and Therapeutic Resistance of Pancreatic Ductal Adenocarcinomas. Biochim. Biophys. Acta Rev. Cancer.

[131] Xu X., Kobayashi S., Qiao W., Li C., Xiao C., Radaeva S., Stiles B., Wang R.H., Ohara N., Yoshino T. (2006). Induction of Intrahepatic Cholangiocellular Carcinoma by Liver-Specific Disruption of Smad4 and Pten in Mice. J. Clin. Investig..

[132] Wu R.C., Wang T.L., Shih Ie M. (2014). The Emerging Roles of ARID1A in Tumor Suppression. Cancer Biol. Ther..

[133] Trotman L.C., Pandolfi P.P. (2003). PTEN and p53: Who will Get the Upper Hand?. Cancer Cell.

[134] Pappas K., Xu J., Zairis S., Resnick-Silverman L., Abate F., Steinbach N., Ozturk S., Saal L.H., Su T., Cheung P. (2017). p53 Maintains Baseline Expression of Multiple Tumor Suppressor Genes. Mol. Cancer Res..

[135] Morran D.C., Wu J., Jamieson N.B., Mrowinska A., Kalna G., Karim S.A., Au A.Y., Scarlett C.J., Chang D.K., Pajak M.Z. (2014). Targeting mTOR Dependency in Pancreatic Cancer. Gut.

[136] Revia S., Seretny A., Wendler L., Banito A., Eckert C., Breuer K., Mayakonda A., Lutsik P., Evert M., Ribback S. (2022). Histone H3K27 Demethylase KDM6A is an Epigenetic Gatekeeper of mTORC1 Signalling in Cancer. Gut.

[137] Hassan Z., Schneeweis C., Wirth M., Veltkamp C., Dantes Z., Feuerecker B., Ceyhan G.O., Knauer S.K., Weichert W., Schmid R.M. (2018). MTOR Inhibitor-Based Combination Therapies for Pancreatic Cancer. Br. J. Cancer.

[138] Belli C., Repetto M., Anand S., Porta C., Subbiah V., Curigliano G. (2023). The Emerging Role of PI3K inhibitors For Solid Tumour Treatment And Beyond. Br. J. Cancer.

[139] Pervanidis K.A., D’Angelo G.D., Weisner J., Brandherm S., Rauh D. (2024). Akt Inhibitor Advancements: From Capivasertib Approval to Covalent-Allosteric Promises. J. Med. Chem..

[140] Chen L.J., Xu X.Y., Zhong X.D., Liu Y.J., Zhu M.H., Tao F., Li C.Y., She Q.S., Yang G.J., Chen J. (2023). The Role of Lysine-Specific Demethylase 6A (KDM6A) in Tumorigenesis and its Therapeutic Potentials in Cancer therapy. Bioorg. Chem..

[141] Hirt C.K., Booij T.H., Grob L., Simmler P., Toussaint N.C., Keller D., Taube D., Ludwig V., Goryachkin A., Pauli C. (2022). Drug Screening and Genome Editing in Human Pancreatic Cancer Organoids Identifies Drug-Gene Interactions and Candidates for Off-Label Treatment. Cell Genom..

[142] Botta G.P., Kato S., Patel H., Fanta P., Lee S., Okamura R., Kurzrock R. (2021). SWI/SNF complex alterations as a biomarker of immunotherapy efficacy in pancreatic cancer. JCI Insight.

[143] Tomihara H., Carbone F., Perelli L., Huang J.K., Soeung M., Rose J.L., Robinson F.S., Lissanu Deribe Y., Feng N., Takeda M. (2021). Loss of ARID1A Promotes Epithelial-Mesenchymal Transition and Sensitizes Pancreatic Tumors to Proteotoxic Stress. Cancer Res..

